# Transcriptional response of the heart to vagus nerve stimulation

**DOI:** 10.1152/physiolgenomics.00095.2023

**Published:** 2023-12-04

**Authors:** Daniel O. Kellett, Qadeer Aziz, Jonathan D. Humphries, Alla Korsak, Alice Braga, Ana Gutierrez Del Arroyo, Marilena Crescente, Andrew Tinker, Gareth L. Ackland, Alexander V. Gourine

**Affiliations:** ^1^Centre for Cardiovascular and Metabolic Neuroscience, Department of Neuroscience, Physiology and Pharmacology, University College London, London, United Kingdom; ^2^Translational Medicine and Therapeutics, William Harvey Research Institute, Queen Mary University of London, London, United Kingdom; ^3^Department of Life Sciences, Manchester Metropolitan University, Manchester, United Kingdom

**Keywords:** autonomic nervous system, heart, RNA sequencing, transcriptome, vagus nerve

## Abstract

Heart failure is a major clinical problem, with treatments involving medication, devices, and emerging neuromodulation therapies such as vagus nerve stimulation (VNS). Considering the ongoing interest in using VNS to treat cardiovascular disease, it is important to understand the genetic and molecular changes developing in the heart in response to this form of autonomic neuromodulation. This experimental animal (rat) study investigated the immediate transcriptional response of the ventricular myocardium to selective stimulation of vagal efferent activity using an optogenetic approach. Vagal preganglionic neurons in the dorsal motor nucleus of the vagus nerve were genetically targeted to express light-sensitive chimeric channelrhodopsin variant ChIEF and stimulated using light. RNA sequencing of the left ventricular myocardium identified 294 differentially expressed genes (false discovery rate < 0.05). Qiagen Ingenuity Pathway Analysis (IPA) highlighted 118 canonical pathways that were significantly modulated by vagal activity, of which 14 had a *z* score of ≥2/≤−2, including EIF-2, IL-2, integrin, and NFAT-regulated cardiac hypertrophy. IPA revealed the effect of efferent vagus stimulation on protein synthesis, autophagy, fibrosis, autonomic signaling, inflammation, and hypertrophy. IPA further predicted that the identified differentially expressed genes were the targets of 50 upstream regulators, including transcription factors (e.g., MYC and NRF1) and microRNAs (e.g., miR-335-3p and miR-338-3p). These data demonstrate that the vagus nerve has a major impact on the myocardial expression of genes involved in the regulation of key biological pathways. The transcriptional response of the ventricular myocardium induced by stimulation of vagal efferents is consistent with the beneficial effect of maintained/increased vagal activity on the heart.

**NEW & NOTEWORTHY** This experimental animal study investigated the immediate transcriptional response of the ventricular myocardium to selective stimulation of vagal efferent activity. Vagal stimulation induced significant transcriptional changes in the heart involving the pathways controlling autonomic signaling, inflammation, fibrosis, and hypertrophy. This study provides the first direct evidence that myocardial gene expression is modulated by the activity of the autonomic nervous system.

## Graphical abstract

**Figure:**
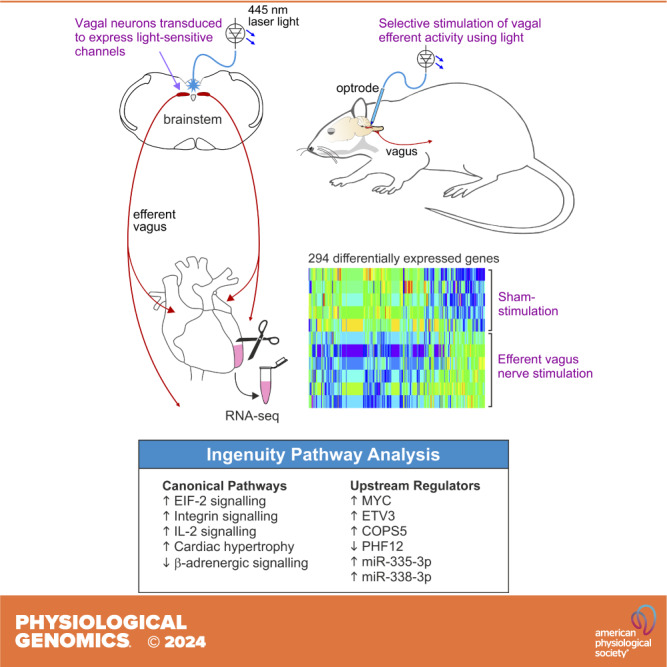

## INTRODUCTION

Heart failure is a major clinical and public health burden, characterized by its high prevalence, high morbidity, and poor prognosis. Clinical treatments include medication such as diuretics, β-adrenoceptor antagonists, angiotensin-converting enzyme inhibitors, and mineralocorticoid receptor antagonists (1, 2). In some patients, device-based treatments have been shown to be effective, such as cardiac resynchronization therapy (3), and cardiac contractility modulation (4, 5). In severe cases of heart failure, left ventricular assist devices are used (6), and ultimately heart transplantation is required in refractory cases (7). In the last decade, vagus nerve stimulation (VNS) has emerged as a potential therapy for chronic heart failure (8–10).

VNS is a Federal Drug Administration-approved treatment for drug-resistant epilepsy (11–13) and depression (14). Animal studies using experimental models of heart failure have demonstrated that VNS is effective in preventing ventricular fibrillation (15), reducing sudden cardiac death (16), attenuating cardiac remodeling and systemic inflammation (17), as well as improving left ventricular function (18). In a rat model of chronic heart failure, VNS was shown to markedly improve survival (19).

The first human trials of VNS in heart failure were single-center (20) and multi-center studies (21) that used stimulation electrodes designed for preferential activation of vagal efferent (motor) fibers, using cathodic induction with simultaneous asymmetrical anodal blocks (20). These trials showed that VNS improves quality of life and some parameters of cardiac function. Subsequently, three randomized controlled trials have been completed: NECTAR-HF (22, 23), INOVATE-HF (24), and ANTHEM-HF (25). The ANTHEM-HF trial showed long-term improvements in measures including baseline heart rate, heart rate variability, and left ventricular end-systolic volume (25), but the other two trials failed to meet primary endpoints, although some secondary outcomes were met, such as improvements in quality of life. The ANTHEM-HFrEF Pivotal Study is currently ongoing (26).

Considering the significant interest in using VNS to treat cardiovascular disease (27–29) it is important to understand the genetic and molecular changes developing in the heart in response to this form of autonomic neuromodulation. Parasympathetic control of the heart is provided by two populations of vagal preganglionic neurons that reside in the lower brain stem: the dorsal motor nucleus of the vagus nerve (DVMN) and the nucleus ambiguus (30–32). There is significant evidence that chronotropic control of the heart is provided predominantly by the neurons of the nucleus ambiguus which synapse on neurons of the intrinsic cardiac ganglia innervating the sinoatrial node, whilst cardiac DVMN neurons control the activity of postganglionic projections that innervate the ventricular myocardium and modulate ventricular excitability and contractility (30, 32). In this experimental animal study conducted in rats, we investigated the immediate transcriptional response of the ventricular myocardium to selective stimulation of vagal preganglionic neurons of the DVMN using an optogenetic approach. We hypothesized that stimulation of vagal efferent activity would trigger a transcriptional response which would be consistent with the beneficial effect of VNS on the heart.

## MATERIALS AND METHODS

All the experiments were performed in accordance with the European Commission Directive 2010/63/EU (European Convention for the Protection of Vertebrate Animals used for Experimental and Other Scientific Purposes) and the UK Home Office Animals (Scientific Procedures) Act (1986) with project approval from the UCL Institutional Animal Care and Use Committee. Rats were group-housed and maintained on a 12:12-h light-dark cycle (lights on 0700) with ad libitum access to food and water.

### Optogenetic Stimulation of Vagal Efferent Activity

**Figure 1.**
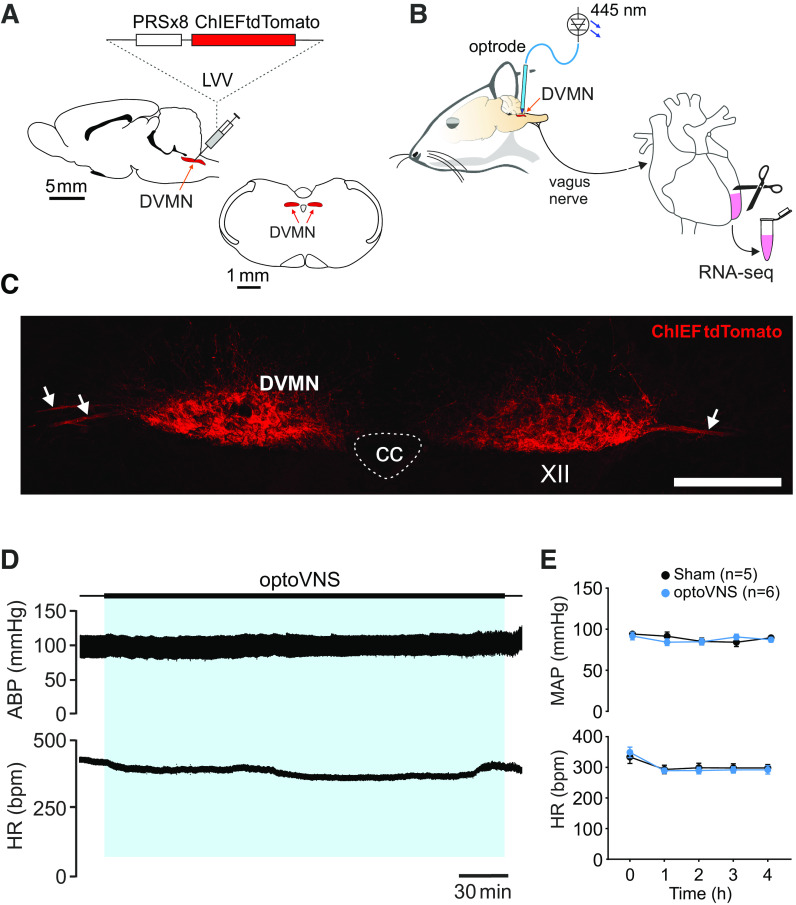
Genetic targeting of vagal preganglionic neurons in the dorsal motor nucleus of the vagus nerve (DVMN) to express light-sensitive channel ChIEF for selective stimulation of vagal efferent activity using light. *A*: the rat brain drawn in sagittal and coronal projections illustrating the anatomic location of the DVMN targeted with a lentiviral vector (LVV) to express ChIEFtdTomato under the control of the PRSx8 promoter. *B*: schematic drawing of the experimental design in anesthetized rats instrumented for stimulation of the DVMN neurons expressing ChIEF by application of 445-nm laser light followed by RNA sequencing of the left ventricular myocardium. *C*: photomicrograph of a coronal section of the rat dorsal brain stem taken at low magnification illustrating a representative example of ChIEFtdTomato expression in the caudal regions of the DVMN. Arrows point at projecting axons of the transduced DVMN neurons, forming the efferent vagus nerve. XII, hypoglossal nucleus; CC, central canal. Scale bar = 200 μm. *D*: representative recordings illustrating changes in arterial blood pressure (ABP) and heart rate (HR) during 4 h of DVMN stimulation [optical vagus nerve stimulation (optoVNS); 5 Hz] in an isoflurane-anesthetized rat. *E*: mean (±SE) values of mean arterial blood pressure (MAP) and HR at baseline (0) and at 1, 2, 3, and 4 h of optoVNS or sham stimulation in isoflurane-anesthetized rats. Stimulation of DVMN neurons at 5 Hz had no effect on ABP and HR.

Vagal preganglionic neurons of the DVMN were targeted to express a light-sensitive chimeric channelrhodopsin variant ChIEF fused with a fluorescent protein tdTomato (ChIEFtdTomato) or enhanced green fluorescent protein (eGFP; control transgene) using lentiviral vectors (LVV) (Fig. 1*A*). Transgene expression was driven under the control of a Phox2-activated promoter PRSx8 (33). Validation of the specificity of vectors in transducing DVMN neurons was described in detail previously (34, 35). Pulses of blue (445 nm) light trigger precisely timed depolarizations and action potential firing of DVMN neurons transduced to express ChIEF (35) and induce robust increases in vagal efferent activity (36, 37).

Sixteen male Sprague–Dawley rats (150–200 g, 5–6 wk of age, RRID:RRRC_00239) were anesthetized with a combination of ketamine (60 mg·kg^−1^ im) and medetomidine (250 µg·kg^−1^ im). Adequate depth of surgical anesthesia was maintained and confirmed by the absence of a withdrawal response to a paw pinch. The head of the animal was fixed in a stereotaxic frame and the dorsal brain stem surface was exposed via a midline dorsal neck incision, followed by a small transverse opening of the atlanto-occipital membrane. LVV-PRSx8-ChIEFtdTomato or LVV-PRSx8-eGFP (control vector) were microinjected into the DVMN using glass micropipettes. Bilateral microinjections of the vectors (0.5 µL at a rate of 0.1 µL·min^−1^) were made at 0.5 mm rostral, 0.6 mm lateral, and 0.8 mm ventral from the surface of the brain stem, measured after the initial positioning of the microinjection pipette tip at calamus scriptorius as a visual landmark. After the injections, the wound was sutured and anesthesia was reversed with atipamezole (1 mg·kg^−1^ im). Buprenorphine (0.05 mg·kg^−1^ per day, sc) was administered postoperatively for 3 days after surgery. There were no postoperative complications; the animals recovered and gained weight normally, with no attrition over the course of the study.

After 4 wk [to allow high and stable transgene expression in the DVMN (34, 38)], animals were anesthetized with isoflurane (induction: 5%; maintenance: 2–2.5% in 1:1 O_2_/air mixture). Volatile anesthesia was chosen for the main experiment as isoflurane was expected to dissipate rapidly from the tissue when the heart was removed for collection of the left ventricular sample. The animal’s head was fixed in a stereotaxic frame. Body temperature was maintained at 37.0 ± 0.5°C via a homeothermic heating pad. A polythene cannula filled with heparinized saline was placed in the left femoral artery for the recording of arterial blood pressure (ABP); heart rate (HR) was derived from the ABP waveform using Spike2 software (CED, Cambridge, UK, RRID:SCR_000903). Adequate depth of anesthesia was monitored by the stability of ABP and HR recordings in response to a paw pinch. The dorsal surface of the brain stem was exposed by opening the atlanto-occipital membrane and removing part of the occipital bone to aid the placement of the optrode for light delivery to the DVMN bilaterally (Fig. 1*B*).

After at least 15 min of baseline ABP and HR had been recorded, optogenetic stimulation (10–20 ms of 445 nm light pulses at 5 Hz for 4 h) was applied to the dorsal brain stem to stimulate DVMN neurons expressing ChIEFtdTomato (*n* = 8) (Fig. 1*B*). We call this treatment optical VNS (optoVNS). Animals transduced to express eGFP in the DVMN received the same light pulses applied to the brain stem (sham stimulation protocol; *n* = 8). Previous experiments have shown that efferent vagus nerve activity increases in response to light pulses only in animals transduced to express ChIEFtdTomato in the DVMN (36, 37). Mean resting discharge frequency of the DVMN neurons recorded in sedentary rats under anesthesia is ∼2.5 Hz (39). In the present study optoVNS was applied with a frequency of 5 Hz to mimic the physiological increases in discharge of the DVMN neurons recorded during acute exercise and after exercise training (39). A period of 4 h of stimulation was selected to study the acute transcriptional response of the myocardium induced by increased vagal efferent activity.

### Sample Collection, RNA Extraction, and Histology

Following the period of stimulation, animals were given an overdose of isoflurane, and the hearts were rapidly removed and rinsed in ice-cold PBS. The left ventricle was quickly dissected and snap-frozen in liquid nitrogen. The brains were removed and fixed in 4% phosphate-buffered paraformaldehyde (pH 7.4). After cryoprotection in 30% sucrose, the brain stem was isolated, and a sequence of coronal sections (30 μm) was cut on a cryostat. The sections were mounted on glass slides and examined using a fluorescence microscope to determine the extent of ChiEFtdTomato or eGFP expression in the DVMN.

Total RNA was extracted from 30 mg of left ventricular myocardium using an RNeasy mini kit, as per the manufacturer’s instructions. An on-column DNase I treatment (Qiagen) was performed to eliminate genomic DNA contamination. RNA quantity and quality were determined using a Nanodrop1000 spectrophotometer (RRID:SCR_016517) and Agilent Bioanalyzer (RRID:SCR_018043). Only RNA samples of suitable quality were used for RNA sequencing (RNA integrity > 7, rRNA ratio 28 s/18s > 2, and concentration greater than 10 ng/µL).

### RNA Sequencing and Differential Gene Expression Analysis

RNA sequencing was performed by the Queen Mary University of London Genome Center (40). RNA libraries were generated with TruSeq Stranded Total RNA library prep with RiboZero (Illumina) with >100 million short reads generated per sample. The quality of the sequencing reads (fastq files) was assessed by FastQC (version 0.11.2, RRID:SCR_014583). The reads were trimmed for adaptor sequences and poor-quality reads with Trim Galore (version 0.3.7, RRID:SCR_011847). Two trimming phases were applied, the first to remove adaptors and the second to remove poly G sequences. The quality of the trimmed sequences was reassessed with FastQC (version 0.11.2). After quality control, trimmed sequences were aligned to the coding regions of the reference genome (Rn7) with STAR (version 2.3.7a, RRID:SCR_004463) and Bowtie2 (version 2.2.3, RRID:SCR_016368). Transcript abundance was then calculated by HTSeq-counts software (version 0.6.0, RRID:SCR_005514) (41). Differential expression analysis was performed using Partek Genomics Suite (RRID:SCR_011860) Gene Specific Analysis (GSA). Genes with low counts (<10 cumulatively across all samples) were excluded from the analysis. Gene expression was normalized using the DESeq2 (RRID:SCR_015687) median of ratios normalization. Differentially expressed genes (DEGs) were identified with a false discovery rate (FDR) of <0.05. FDR represents the expected proportion of falsely identified DEGs. Principal component analysis (PCA) of principal components 1 and 2 was performed in Partek GSA; PCA, volcano, and heatmap plots were generated in R.

### Ingenuity Pathway Analysis

To gain insight into the canonical pathways and upstream regulators modulated by vagal activity, DEGs were further analyzed using the Ingenuity Pathway Analysis (IPA) platform [QIAGEN (42), RRID:SCR_008653]. The analysis was conducted using IPA default settings, with an FDR of <0.05 and fold change used to calculate IPA analysis-ready molecules, and *z* scores, representing the activation state of each canonical pathway or upstream regulator (*z* score ≥ 2 significantly activated and *z* score ≤ −2 significantly inhibited).

The Canonical Pathways Analysis module of IPA was used to identify biological pathways that were predicted to be activated or inhibited, based on significant enrichment of data within the DEG data set (*P* < 0.05 and *z* score ≥2/≤−2). Additional pathways were also explored that were significantly enriched but had lower *z* scores or had no predicted *z* scores.

The Upstream Regulator Analysis module of IPA was used to identify the upstream molecules that could explain the observed changes in gene expression. These in turn could help to understand the major transcriptional changes that develop in the ventricular myocardium in response to optoVNS. A *P* value of <0.05 and a *z* score of ≥2/≤−2 were used to identify upstream regulators that were predicted to be significantly enriched. The algorithms used to calculate *P* values of overlap and *z* scores in IPA have been previously described in detail (42).

### Analysis of Cardiovascular Variables

Mean arterial blood pressure (MAP) and HR values were derived from the recorded ABP waveform. MAP and HR values were averaged over a period of 60 s at baseline (before optoVNS or sham stimulation) and at 1, 2, 3, and 4 h after the onset of stimulation. Results were analyzed by two-way ANOVA with repeated measures using R. Results are expressed as means ± SE.

### Data Analysis and Visualization

Animals were randomly assigned unique identification numbers, which were known to the researchers performing microinjection surgeries and optoVNS experiments, but not to the investigators who conducted sequencing and subsequent data analysis until raw counts had been calculated. This protocol ensured that the investigators who conducted the gene expression analysis remained blinded to the experimental group assignments until after the data collection. An initial group size of eight animals per study group was considered sufficient, based on the data obtained in similar types of the experiments involving measurements of cardiovascular variables and individual gene analysis in healthy laboratory rats (39, 43).

Raw counts were processed into differential data by Partek GSA, and subsequently submitted for IPA using a criterion of FDR < 0.05. Raw count data were analyzed in Partek GSA and visualized in GraphPad Prism (RRID:SCR_002798). Chord diagrams were generated using the circlize package in R (RRID:SCR_002141) (44).

## RESULTS

Of the eight animals that received sham stimulation, one animal was excluded due to persistent cardiac arrhythmia over the course of the experiment, one animal due to lack of eGFP expression in the DVMN, and one sample failed the initial library quality control. Of the eight animals given optoVNS, one animal was excluded due to the expression of the transgene in the neighboring nuclei, and one sample failed the initial library quality control. This yielded final experimental group sizes of *n* = 6 (optoVNS) and *n* = 5 (sham) for DEG and IPA analysis.

Baseline MAP values were 92 ± 5 and 94 ± 2 mmHg, and baseline HR values were 348 ± 17 and 333 ± 21 beats/min in the optoVNS (*n* = 6) and sham (*n* = 5) groups, respectively. MAP and HR changes at 1–4 h after the onset of light stimulation were analyzed by two-way ANOVA (with time as the repeated measure). Over the course of the experiment, there were no significant differences in MAP [*F*_(1,49)_ = 0.884, *P* = 0.352] or HR [*F*_(1,49)_ = 0.832, *P* = 0.336] between the groups of animals that received optoVNS and sham stimulation (Fig. 1*E*).

### Histology

Coronal brain stem sections were inspected under a fluorescence microscope (45). The expression of ChIEFtdTomato was confirmed by red fluorescence (Fig. 1*C*) and expression of eGFP by green fluorescence, as described previously (34, 37, 38, 43). A high level of transgene expression in DVMN neurons was observed in all the animals used for the analysis of myocardial gene expression.

### RNA-Seq and Analysis of DEGs

Analysis of transcripts from the left ventricular tissue of six experimental (optoVNS) and five control (sham-stimulated) animals identified 13,525 genes, of which 2,391 were excluded due to low counts (defined as cumulative counts of <10 across all samples), leaving 11,134 genes for the subsequent analysis of DEGs. The analysis revealed 2,632 DEGs (23%) with an uncorrected *P* < 0.05, of which 62% were upregulated and 38% downregulated. Two hundred ninety-four genes satisfied FDR < 0.05, of which 235 were upregulated and 59 downregulated (80% and 20% respectively; see Supplemental Table S1 for the full listing of DEGs with FDR < 0.05 and Table 1 and Table 2 for the top 20 DEGs that were found to be upregulated and downregulated, respectively).

**Table 1.** Top DEGs (upregulated)

	Symbol	Full Name	*P*	FDR	FC
1	*Fkbp3*	FKBP prolyl isomerase 3	8.15 *e*^−6^	2.53 *e*^−2^	1.51
2	*Usp33*	Ubiquitin-specific peptidase 33	1.43 *e*^−5^	2.53 *e*^−2^	1.38
3	*Arhgap5*	Rho GTPase activating protein 5	1.63 *e*^−5^	2.53 *e*^−2^	1.54
4	*Cul3*	Cullin 3	1.83 *e*^−5^	2.53 *e*^−2^	1.34
5	*Pip4p2*	Phosphatidylinositol 4,5-bisphosphate 4-phosphatase 2	2.02 *e*^−5^	2.53 *e*^−2^	1.67
6	*Kpna4*	Karyopherin subunit α4	2.08 *e*^−5^	2.53 *e*^−2^	1.49
7	*Prkaa1*	Protein kinase AMP-activated catalytic subunit α1	2.22 *e*^−5^	2.53 *e*^−2^	1.47
8	*Zfp91*	Zinc finger protein, atypical E3 ubiquitin ligase	2.27 *e*^−5^	2.53 *e*^−2^	1.31
9	*Ube3a*	Ubiquitin protein ligase E3A	3.17 *e*^−5^	2.79 *e*^−2^	1.35
10	*Aff4*	ALF transcription elongation factor 4	3.74 *e*^−5^	2.79 *e*^−2^	1.25
11	*Rdx*	Radixin	4.32 *e*^−5^	2.79 *e*^−2^	1.29
12	*Tpp2*	Tripeptidyl peptidase 2	4.39 *e*^−5^	2.79 *e*^−2^	1.34
13	*Rps8*	Ribosomal protein S8	4.89 *e*^−5^	2.79 *e*^−2^	1.34
14	*Marchf7*	Membrane-associated ring-CH-type finger 7	4.90 *e*^−5^	2.79 *e*^−2^	1.35
15	*Jam2*	Junctional adhesion molecule 2	5.06 *e*^−5^	2.79 *e*^−2^	1.29
16	*Cycs*	Cytochrome *c*, somatic	5.34 *e*^−5^	2.79 *e*^−2^	1.56
17	*Fcho2*	FCH and μ domain containing endocytic adaptor 2	5.34 *e*^−5^	2.79 *e*^−2^	1.41
18	*Ralgapa1*	Ral GTPase activating protein catalytic subunit α1	5.65 *e*^−5^	2.79 *e*^−2^	1.25
19	*Zfp655*	Zinc finger protein 655	6.05 *e*^−5^	2.79 *e*^−2^	1.37
20	*Lpgat1*	Lysophosphatidylglycerol acyltransferase 1	6.26 *e*^−5^	2.79 *e*^−2^	1.32
21	*Zfp68*	Zinc finger protein 68	6.56 *e*^−5^	2.79 *e*^−2^	2.06
22	*Usp9x*	Ubiquitin-specific peptidase 9 X-linked	7.74 *e*^−5^	2.79 *e*^−2^	1.38
23	*Ppp4r3b*	Protein phosphatase 4 regulatory subunit 3B	8.41 *e*^−5^	2.79 *e*^−2^	1.33
24	*Clock*	Clock circadian regulator	1.08 *e*^−4^	2.79 *e*^−2^	2.01
25	*Eif3a*	Eukaryotic translation initiation factor 3 subunit A	1.52 *e*^−4^	2.79 *e*^−2^	1.29
26	*Cul5*	Cullin 5	1.93 *e*^−4^	2.93 *e*^−2^	1.37
27	*Lactb2*	Lactamase β2	2.22 *e*^−4^	3.09 *e*^−2^	1.37
28	*Atf2*	Activating transcription factor 2	2.97 *e*^−4^	3.41 *e*^−2^	1.33
29	*Trpm7*	Transient receptor potential cation channel subfamily M member 7	3.15 *e*^−4^	3.48 *e*^−2^	1.29
30	*Eif5*	Eukaryotic translation initiation factor 5	4.36 *e*^−4^	3.82 *e*^−2^	1.39
31	*Cul2*	Cullin 2	5.08 *e*^−4^	3.82 *e*^−2^	1.37
32	*Cul1*	Cullin 1	5.72 *e*^−4^	3.82 *e*^−2^	1.26
33	*Wnk1*	WNK lysine deficient protein kinase 1	6.14 *e*^−4^	3.82 *e*^−2^	2.63
34	*Tmem33*	Transmembrane protein 33	6.28 *e*^−4^	3.83 *e*^−2^	1.46
35	*Eif4g2*	Eukaryotic translation initiation factor 4 γ 2	7.32 *e*^−4^	3.98 *e*^−2^	1.28
36	*Slc8a1*	Solute carrier family 8 member A1	1.02 *e*^−3^	4.36 *e*^−2^	1.43
37	*Zmynd11*	Zinc finger MYND-type containing 11	1.04 *e*^−3^	4.36 *e*^−2^	2.23
38	*Golga5*	Golgin A5	1.11 *e*^−3^	4.42 *e*^−2^	1.32

Upregulated differentially expressed genes [DEGs; false discovery rate (FDR) < 0.05]. Top 20 genes plus 18 additional genes of interest are listed. See Supplemental Table S1 for a full list. *P*, uncorrected *P* value; FC, fold change (experimental vs. control).

**Table 2.** Top DEGs (downregulated)

	Symbol	Full Name	*P*	FDR	FC
1	*Col1a2*	Collagen type I α_2_ chain	1.82 *e*^−5^	2.53 *e*^−2^	−1.40
2	*Trim72*	Tripartite motif containing 72	9.42 *e*^−6^	2.53 *e*^−2^	−1.27
3	*Crtapl1*	Cartilage-associated protein-like 1	1.31 *e*^−4^	2.79 *e*^−2^	−2.30
4	*RT1-S3*	RT1 class Ib, locus S3	1.51 *e*^−4^	2.79 *e*^−2^	−1.81
5	*Hist2h4*	H4 clustered histone 14	1.51 *e*^−4^	2.79 *e*^−2^	−1.53
5	*Axl*	AXL receptor tyrosine kinase	1.38 *e*^−4^	2.79 *e*^−2^	−1.52
6	*Chpf2*	Chondroitin polymerizing factor 2	1.11 *e*^−4^	2.79 *e*^−2^	−1.44
7	*Col1a1*	Collagen type I α_1_ chain	1.49 *e*^−4^	2.79 *e*^−2^	−1.52
7	*Cbx6*	Chromobox 6	1.42 *e*^−4^	2.79 *e*^−2^	−1.40
8	*Rnf187*	Ring finger protein 187	1.53 *e*^−4^	2.79 *e*^−2^	−1.22
9	*Rbck1*	RANBP2 type and C3HC4 type zinc finger containing 1	1.92 *e*^−4^	2.93 *e*^−2^	−1.30
10	*Obsl1*	Obscurin-like cytoskeletal adaptor 1	2.10 *e*^−4^	3.00 *e*^−2^	−1.29
11	*Crebzf*	CREB/ATF BZIP transcription factor	2.48 *e*^−4^	3.14 *e*^−2^	−2.70
12	*Notch4*	Notch receptor 4	2.54 *e*^−4^	3.14 *e*^−2^	−1.66
13	*Igfbp7*	Insulin-like growth factor binding protein 7	2.74 *e*^−4^	3.31 *e*^−2^	−1.44
14	*Cntfr*	Ciliary neurotrophic factor receptor	3.10 *e*^−4^	3.46 *e*^−2^	−1.85
15	*Plec*	Plectin	3.66 *e*^−4^	3.82 *e*^−2^	−1.63
16	*Socs1*	Suppressor of cytokine signaling 1	5.27 *e*^−4^	3.82 *e*^−2^	−2.79
17	*Fcgbp*	Fcγ binding protein	5.43 *e*^−4^	3.82 *e*^−2^	−2.23
18	*Exosc4*	Exosome component 4	4.29 *e*^−4^	3.82 *e*^−2^	−1.47
19	*Jun*	Jun proto-oncogene, AP-1 transcription factor subunit	5.21 *e*^−4^	3.82 *e*^−2^	−1.36
20	*Lrp1*	LDL receptor-related protein 1	4.63 *e*^−4^	3.82 *e*^−2^	−1.31
21	*Cygb*	Cytoglobin	4.46 *e*^−4^	3.82 *e*^−2^	−1.25
22	*Col5a1*	Collagen type V α_1_ chain	6.50 *e*^−4^	3.87 *e*^−2^	−1.28
23	*Ifi27l2b*	Interferon, α-inducible protein 27 like 2B	8.53 *e*^−4^	4.15 *e*^−2^	−2.29
24	*Nkx2-5*	NK2 homeobox 5	1.02 *e*^−3^	4.36 *e*^−2^	−1.49
25	*Slc22a17*	Solute carrier family 22 member 17	1.12 *e*^−3^	4.43 *e*^−2^	−1.41
26	*Trim21*	Tripartite motif containing 21	1.19 *e*^−3^	4.50 *e*^−2^	−1.98

Downregulated differentially expressed genes [DEGs; false discovery rate (FDR) < 0.05]. Top 20 genes plus 6 additional genes of interest are listed. See Supplemental Table S1 for a full list. *P*, uncorrected *P* value; FC, fold change (experimental vs. control).

**Figure 2.**
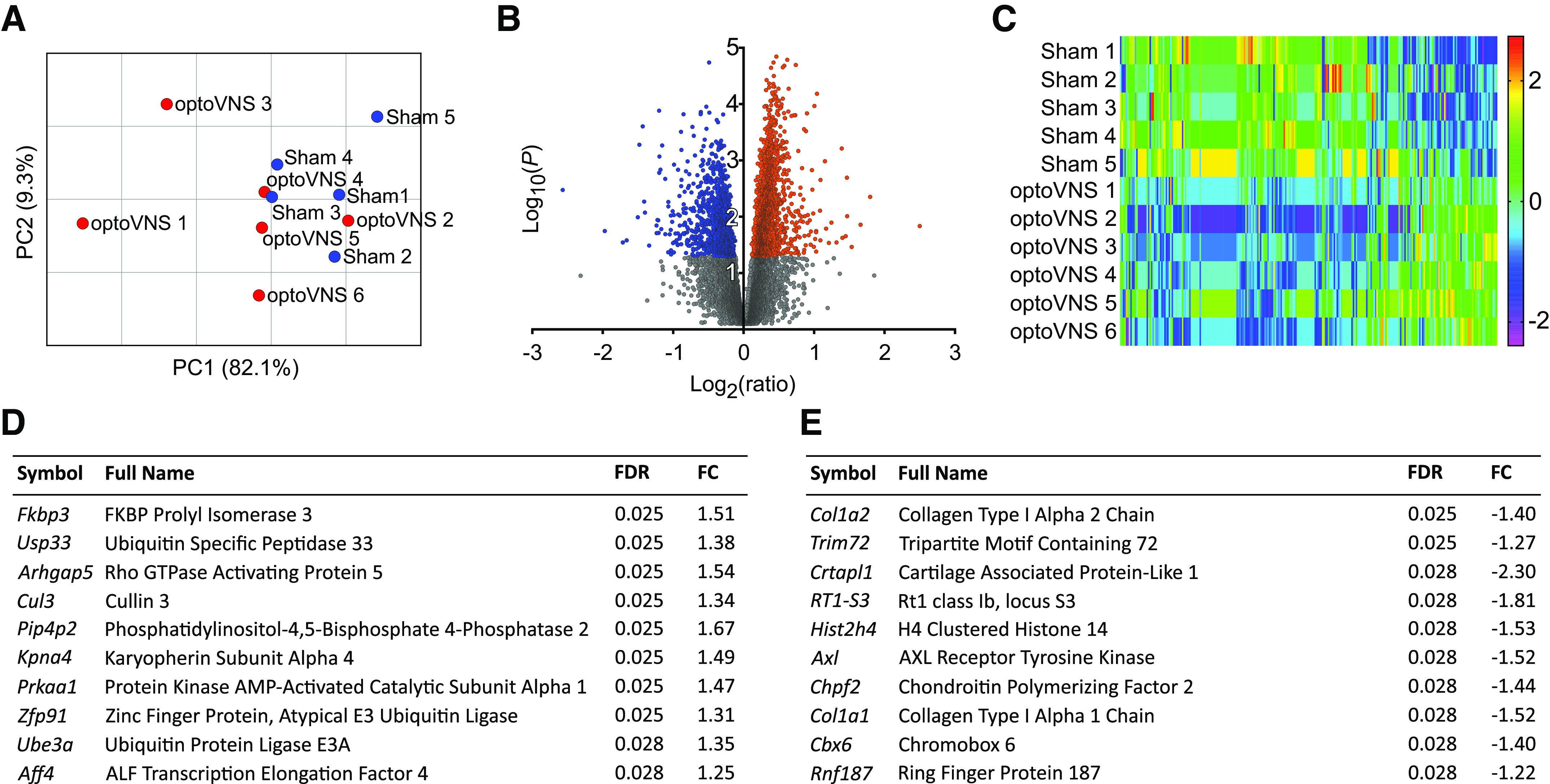
Analysis of differentially expressed genes (DEGs) in the left ventricular myocardium in response to stimulation of vagal efferent activity. *A*: principal component (PC) plot of principal component 1 (PC1) vs. principal component 2 (PC2) of control (sham 1–5) and experimental [optical vagus nerve stimulation (optoVNS) 1–6] samples. *B*: volcano plot of differential expression data of all identified genes. Gray: *P* > 0.05; red: upregulated, *P* < 0.05; blue: downregulated, *P* < 0.05. *C*: heat map of the top 294 DEGs with a false discovery rate (FDR) of <0.05. The scale bar indicates the *z* score. *D*: list of the top 10 most upregulated DEGs. *E*: list of the top 10 most downregulated DEGs. FC, fold change.

PCA plot, volcano plot, and heatmap were used for data visualization (Fig. 2). Principal component 1 (PC1), representing 82.1% of the variance, separated the samples into distinct groups, with the exception of optoVNS sample 2 (Fig. 2*A*). Principal component 2 (PC2), representing a further 9.3% of the variance, did not distinguish between the groups. A volcano plot (Fig. 2*B*) illustrates the distribution of significant (*P* < 0.05) DEGs between log_2_(ratio) −1 to 1, with increasing separation between up- and downregulated DEGs at log_10_(*P*) > 2. A heatmap of the top DEGs (Fig. 2*C*) showed clear separation of the expression data between the groups. OptoVNS sample 2 (an outlier identified by PCA) showed similar expression pattern to other samples in the optoVNS group.

The most significant DEGs are shown in Fig. 2*D* and 2*E*. Among the most significantly upregulated DEGs (by *P* value) were *Usp33, Cul3*, and *Prkaa1* (with fold changes of 1.38, 1.34, and 1.47, respectively). The most significantly downregulated DEGs were *Col1a2, Col1a1, Trim72*, and *Axl* (with fold changes of −1.40, −1.52, −1.27, and −1.52, respectively).

### Ingenuity Pathway Analysis

From the results of previously reported studies involving short periods of daily optoVNS, we were expecting to observe changes in the expression of genes encoding proteins controlling G protein desensitization and recycling (43) as well as genes/pathways related to the regulation of ventricular contractility and excitability as well as activation of cardioprotective mechanisms (34, 37, 38). It was found that optoVNS applied for 4 h had a major impact on the cardiac transcriptome, resulting in the enrichment of pathways related to protein synthesis, ubiquitination and autophagy, autonomic signaling, inflammation, and hypertrophy.

**Figure 3.**
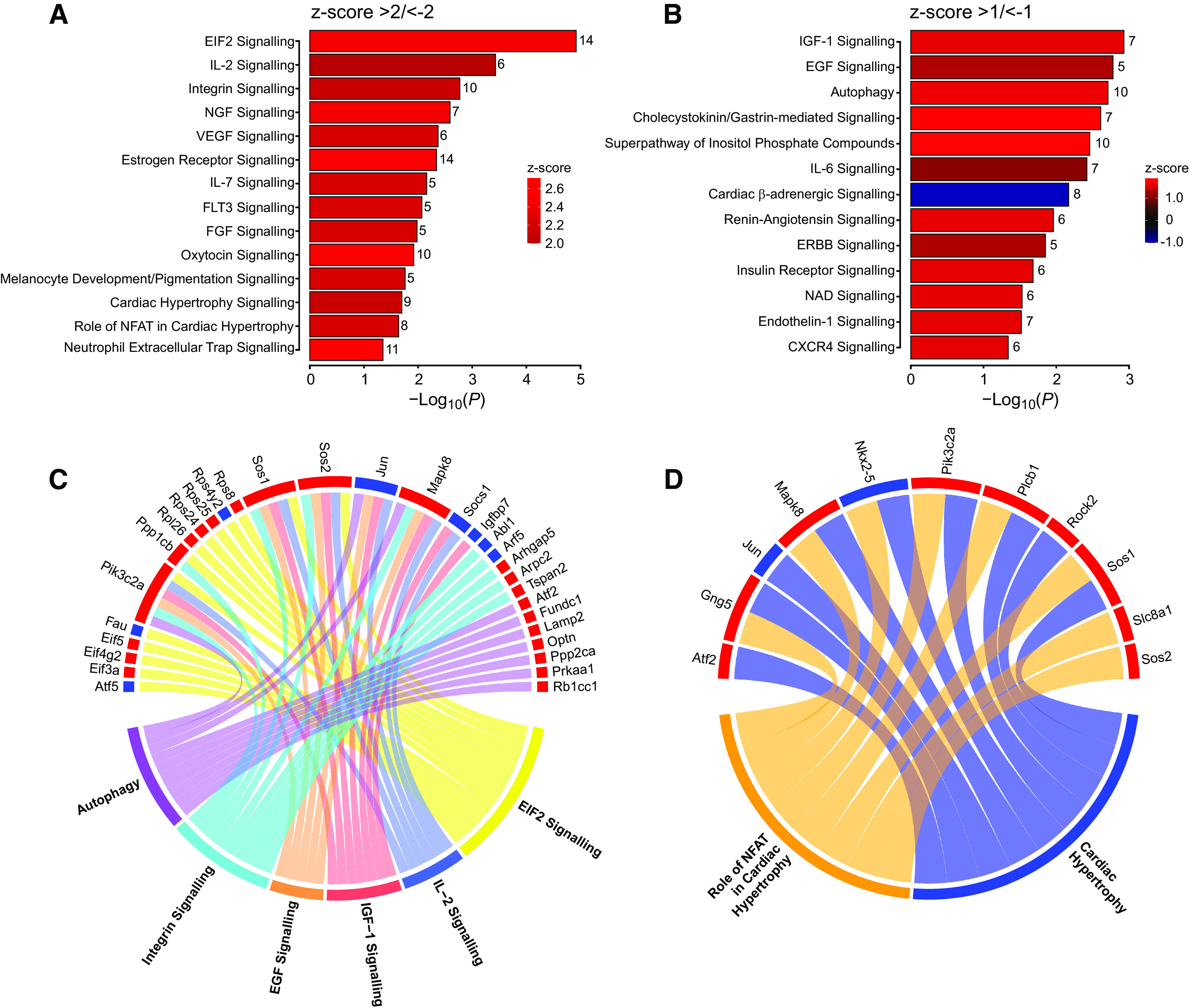
Canonical pathways modulated by vagal efferent activity. *A*: canonical pathways that were significantly enriched in response to optical vagus nerve stimulation (optoVNS; *P* < 0.05, *z* score ≥2/≤−2). *B*: additional canonical pathways of interest (*P* < 0.05, *z* score ≥1/≤−1). The number of overlapping differentially expressed genes (DEGs) from the dataset is shown (full list of genes is provided in Supplemental Table S2). *C*: chord diagram of the six most significantly enriched pathways and their corresponding DEGs. *D*: chord diagram of two cardiac hypertrophy canonical pathways and their corresponding DEGs (red: upregulated, blue: downregulated).

One hundred eighteen significantly enriched canonical pathways were identified by IPA (*P* < 0.05), of which 14 had a *z* score of ≥2/≤−2 (the IPA *z* score algorithm uses the direction and magnitude of gene expression changes within the pathway and provides a standardized measure of predicted activation or inhibition) (Fig. 3*A*). The DEGs involved in these pathways are listed in Supplemental Table S2. The most significantly affected signaling pathways were EIF-2, IL-2, integrin, NGF, VEGF, estrogen receptor, IL-7, FLT3, FGF, oxytocin, cardiac hypertrophy, role of NFAT in cardiac hypertrophy, and neutrophil extracellular trap pathways. Thirteen additional pathways of interest were identified with a *z* score of ≥1/≤−1 and *P* < 0.05 (Fig. 3*B* and Supplemental Table S2). These additional pathways included IGF-1, EGF, autophagy, and CCK/gastrin signaling. The one notable pathway predicted to be inhibited was cardiac β-adrenergic signaling. Finally, pathways with no predicted *z* score were examined. The most significantly enriched of these pathways was the protein ubiquitination pathway [−log_10_(*P*) = 3.48; see Supplemental File FullDataset.xlsx]. The DEGs associated with the most significantly enriched pathways (Supplemental Table S2) were further explored in two chord diagrams. Figure 3*C* shows the six most significantly enriched pathways and the DEGs with which they overlap; Fig. 3*D* shows the two pathways associated with cardiac hypertrophy. In addition, the DEGs most frequently occurring in the predicted pathways were identified: *Pik3c2a* (24/26 pathways), *Sos1* (20/26), *Sos2* (18/26), *Mapk8* (17/26), *Jun* (12/26), and *Atf2* (10/26).

### Upstream Regulators

**Figure 4.**
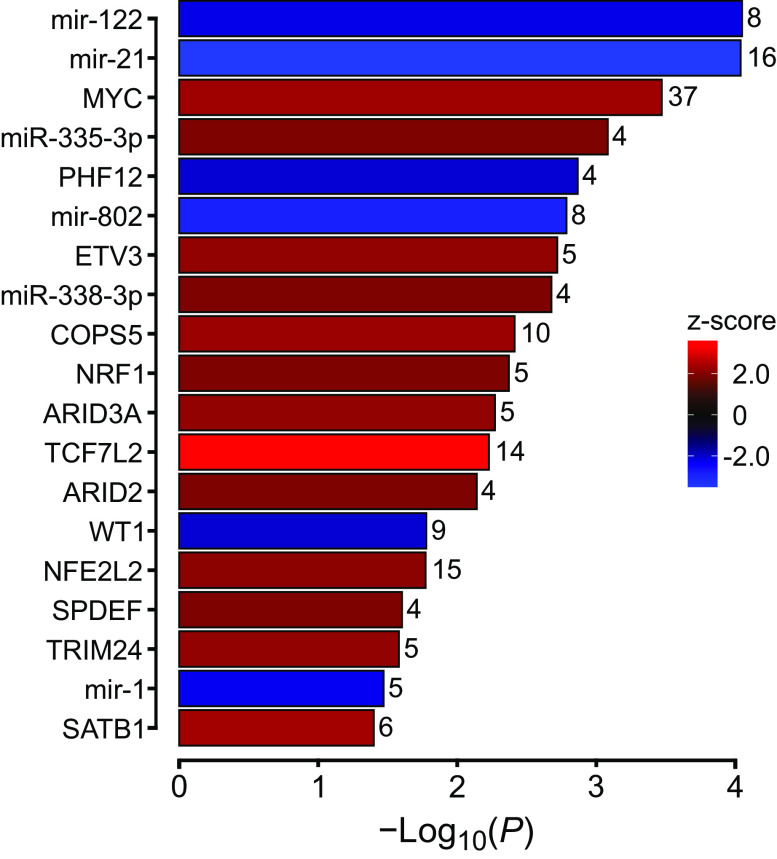
Upstream regulators modulated by vagal efferent activity. Upstream regulators predicted to be activated (positive *z* score) or inhibited (negative *z* score) in response to optical vagus nerve stimulation (optoVNS) are shown (criteria: *P* value of overlap < 0.05, absolute *z* score ≥ 2). mir, microRNA; miR, mature microRNA. The number of overlapping differentially expressed genes are shown (full list of genes is provided in Supplemental Table S3).

Gene expression is regulated by upstream molecules including transcription factors, receptors, kinases, among others. IPA predicted that the DEGs impacted by optoVNS were the targets of 50 upstream regulators, including transcription factors, microRNAs, enzymes, and receptors (*P* value of overlap < 0.05, *z* score ≥2/≤−2). For simplicity, only transcription factors and microRNAs are reported here (19 molecules: see Fig. 4 and Supplemental Table S3). The following transcription factors were predicted to be activated: MYC, ETV3, COPS5, NRF1, ARID3A, TCF7L2, ARID2, NFE2L2, SPDEF, TRIM24, and SATB1. Two transcription factors were predicted to be inhibited: PHF12 and WT1. Four microRNAs were predicted to be inhibited: mir-1, mir-122, mir-21, and mir-802; two microRNAs were predicted to be activated: miR-335-3p and miR-338-3p.

## DISCUSSION

The autonomic nervous system controls the heart by dynamic recruitment and withdrawal of cardiac parasympathetic and sympathetic activities. Vagal efferent projections control heart rate (30), modulate ventricular excitability (38), contractility (34, 46), and protect ventricular cardiomyocytes against ischemia/reperfusion injury (35). The data obtained in this study demonstrate that the vagus nerve has a major impact on myocardial expression of genes involved in regulation of key biological pathways. The transcriptional response of the ventricular myocardium induced by stimulation of vagal efferents is consistent with the beneficial effects of maintained/increased vagal activity on the heart. The full data set (https://doi.org/10.6084/m9.figshare.24449590.v2) provides the readers with the information on genes and pathways modulated by the vagus nerve.

The present study describes early gene expression changes developing in the ventricular myocardium after 4 h of optoVNS. The endpoints of VNS in clinical studies are typically not assessed until weeks or months after the start of the stimulation, therefore, these findings reflect immediate transcriptional changes in the myocardium induced by stimulation of the efferent vagus which are expected to translate into long-term adaptive response. IPA provides insights into the functional implications of such changes by identifying significantly affected biological pathways downstream of early expressed genes.

The observed transcriptional changes show that stimulation of the efferent vagus causes the enrichment of pathways related to protein synthesis, ubiquitination and autophagy, growth and development, autonomic signaling, inflammation, and hypertrophy. Stimulation of vagal efferent activity leads to activation of protein synthesis, as indicated by the enrichment of genes in the EIF-2 signaling pathway. EIF-2 is a key component of the integrated stress response (47). Pathway activation reflects increased transcription and translation, suggesting that optoVNS induces significant gene expression changes in the myocardium. Likewise, protein turnover pathways like ubiquitination and autophagy are activated in response to stimulation, and several ubiquitination-related genes are upregulated (*Usp33* and *Ube3a)*. Ubiquitination removes unwanted proteins and protects the heart against oxidative stress and ischemia; autophagy maintains cardiac energy homeostasis (48) and protects against potential cardiac stress (49), consistent with cardioprotective role of this pathway. Enrichment of pathways involved in growth and development (NGF, VEGF, FGF, and IGF-1) suggests that vagal activity promotes cell growth, tissue development, and angiogenesis.

A number of predicted pathways are related to autonomic signaling. In particular, IPA predicted inhibition of β-adrenergic signaling, although this was not borne out in obvious individual gene expression changes such as altered *Adrb1* expression. One of the DEGs significantly downregulated in this pathway was found to be the ryanodine receptor *Ryr2* (Supplemental Table S1), which plays a major role in the control of cardiac contractility (50), pointing to the molecular basis underlying the negative inotropic influence of cardiac parasympathetic innervation (51).

IPA also predicted the activation of two hypertrophy pathways in response to optoVNS. Cardiac hypertrophy is induced by exercise, pregnancy, and in some pathological conditions to help the heart cope with increased afterload. In cardiovascular disease, such as during the development of hypertension, hypertrophy can become pathological (52). In this process, the nuclear factor of activated T-cells (NFAT) coordinates the signaling pathways associated with pathological remodeling (53). This could explain the enrichment of two proinflammatory pathways: IL-2 and IL-7. Systemic inflammation is also associated with cardiac hypertrophy and could also be a response to early cardiac remodeling induced by VNS (54).

Among the most upregulated DEGs (Fig. 2*D*) were *Usp33, Cul3, Prkaa1*, and *Ube3a*. *Usp33* encodes a deubiquitinase, described as a regulator of β-adrenoceptor signaling, which coordinates receptor recycling and desensitization together with *Usp20* (55). It also interacts with β-arrestin2, causing deubiquitination and β-adrenoceptor internalization (56), which could protect against the high sympathetic activity. β-Arrestin2 was previously shown to be downregulated by optoVNS applied for 15 min per day for 4 days (43). *Cul3* encodes cullin 3, and mutations/disruptions of *Cul3* are associated with familial hyperkalemic hypertension (57), although wider cardiovascular effects have been also suggested (58). *Prkaa1* (also known as *AMPKα1*) encodes a subunit of AMP-activated protein kinase (AMPK), a key regulator of metabolism (59), activated by an increased AMP-to-ATP ratio and facilitating ATP production and autophagy (60, 61). The *Ube3a* gene has recently emerged as a new biomarker of hypertrophy (62), having previously been shown to be associated with myocardial hypertrophy (63).

Among the most downregulated DEGs (Fig. 2*E*) were the collagen type I genes *Col1a1* and *Col1a2*, which encode two α_1_-chains and one α_2_-chain that form the collagen Type I heterotrimer; downregulation of these genes would be expected to slow/disrupt heterotrimer synthesis and reduce collagen deposition. Collagen type I is a marker of cardiac fibrosis (64) and its deposition is inhibited by *Trim72* knockdown, via regulation of Stat3/Notch1 signaling (65). Interestingly, *Trim72* was one of the most downregulated genes identified in the present study, suggesting that vagal activity inhibits collagen deposition and protects against fibrosis. *Axl* (encoding a receptor tyrosine kinase) was also found to be downregulated. In cardiac macrophages, products of *Axl* cross-signal with Tlr4 receptors in response to myocardial ischemia, causing activation of glycolysis, inflammation, and leading to impaired contractility. *Axl* inhibition improves cardiac healing (66), highlighting another potential cardioprotective mechanism recruited by the vagus nerve.

IPA upstream regulator analysis identified several significantly enriched transcription factors and regulators of gene expression, including microRNAs (Fig. 4). MicroRNAs prevent or facilitate the translation of specific mRNA species and are involved in many physiological and pathological processes including modulation of cardiac hypertrophy, fibrosis and apoptosis (67). IPA predicted miR-335-3p and miR-338-3p to be upregulated. Both miR-335-3p and miR-338-3p have been shown to protect the myocardium against ischemia-reperfusion injury (68) and prevent fibrosis by inhibiting cell proliferation and migration of cardiac fibroblasts as well as reducing *Col1a1* expression (69). Both these microRNAs are associated with the downregulation of *Col1a1, Col1a2*, and *Col5a1* genes, further highlighting the beneficial impact of high vagal activity on the heart. MYC and TRIM24, both predicted to be activated by optoVNS, are important cardiac transcription factors involved in cardiac development and both play important roles in modulation of cardiac hypertrophy (70, 71).

### Study Limitations

Previous experimental VNS studies have used a range of electrical stimulation parameters (72). Clinical trials of VNS in heart failure reported mixed efficacy outcomes, possibly reflecting different stimulation protocols (for a review, see Ref. 73). The present study applied optoVNS with a frequency of 5 Hz to mimic the physiological increases in activity of DVMN neurons observed during exercise (39). Unlike VNS used in clinical trials, the stimulation applied in this study was bilateral, but highly specific to efferent projections of the vagus nerve originating from the DVMN.

The long-term effects of increased vagal activity on myocardial gene expression remain to be investigated. In this study, our main objective was to capture the early gene expression changes to gain insights into the immediate transcriptional response of the heart to the efferent VNS. IPA was used to predict early pathway implications, as after 4 h of stimulation the individual gene expression responses may be less pronounced. Future studies will investigate gene expression changes at additional time points, to obtain a broader understanding of the transcriptional response profile over longer durations of stimulation, including chronic VNS.

The experimental approach used in this study, involving optogenetic stimulation of DVMN neurons, effectively recruited not only the vagal efferents projecting to the heart, but also the projections to all other thoracic and abdominal organs that receive parasympathetic innervation. Therefore, the mechanisms responsible for the observed transcriptional changes may include the action of transmitters released by postganglionic cardiac vagal fibers (acetylcholine, nitric oxide, and/or vasoactive intestinal polypeptide) (30) and/or indirect effects due to vagal stimulation-induced release of hormones by the organs of the digestive system (such as somatostatin, Glucagon-Like Peptide 1 and others) entering the systemic circulation and acting on the heart (39). Nonetheless, the data obtained are highly relevant as vagal projections to other organs are also recruited by the VNS devices used in clinical trials.

Another limitation is the use of isoflurane anesthesia, and future studies involving longer periods of stimulation could be performed in awake animals implanted with optic fibers or involving stimulation of transduced projections by application of light to the cervical vagus as described (36).

The method of bulk RNA-sequencing used in this study is more sensitive in detecting changes in overall transcriptional patterns, but does not have the resolution of single-cell RNA-seq. It is important to note that while affording cellular resolution, single-cell RNA-seg is significantly less sensitive in reporting subtle changes in gene expression or expression of less abundant genes. Although there was no present objective to undertake single-cell RNA-seq, this could be explored in future studies to determine the cellular specificity of the observed changes.

### Conclusions

This experimental animal study investigated the immediate transcriptional response of the ventricular myocardium to selective stimulation of vagal efferent activity. The data obtained support the hypothesis that vagal stimulation leads to significant transcriptional changes which may explain the beneficial effects of VNS on the heart. Marked downregulation of collagen genes, along with the related genes (*Trim72*), pathways, and regulators (miR-338-3p) in response to optoVNS was observed, highlighting that the mechanisms underlying the beneficial effects of VNS may include reduction of collagen expression and deposition, crucial factors in cardiac fibrosis, adverse remodeling, and pathological hypertrophy. To the best of our knowledge, this study provides the first direct evidence that myocardial gene expression is modulated by the activity of the autonomic nervous system.

## DATA AVAILABILITY

The data that support the findings of this study are available from the corresponding author upon reasonable request.

## SUPPLEMENTAL DATA

10.6084/m9.figshare.24449590.v2 Supplemental Material and Supplemental Tables S1–S3: https://doi.org/10.6084/m9.figshare.24449590.v2.

## GRANTS

This work was supported by British Heart Foundation Grant RG/19/5/34463. G.L.A. was supported by a British Journal of Anaesthesia/Royal College of Anaesthetists Basic Science Career Development Award and BOC research chair grant in anesthesia from the Royal College of Anaesthetists. This work was facilitated by the NIHR Biomedical Research Centre at Barts. Access to the IPA platform was funded by the Faculty of Biology, Medicine and Health of the University of Manchester.

## DISCLOSURES

No conflicts of interest, financial or otherwise, are declared by the authors.

## AUTHOR CONTRIBUTIONS

A.V.G. conceived and designed research; A.K., A.B., A.G.D.A., and A.V.G. performed experiments; D.O.K., Q.A., J.D.H., A.B., and M.C. analyzed data; D.O.K., Q.A., J.D.H., M.C., A.T., G.L.A., and A.V.G. interpreted results of experiments; D.O.K., Q.A., and J.D.H. prepared figures; D.O.K., Q.A., and A.V.G. drafted manuscript; D.O.K., Q.A., J.D.H., A.K., M.C., A.T., G.L.A., and A.V.G. edited and revised manuscript; all authors revised the article critically for important intellectual content.
